# Prophylactic and Therapeutic EBV Vaccines: Major Scientific Obstacles, Historical Progress, and Future Direction

**DOI:** 10.3390/vaccines9111290

**Published:** 2021-11-07

**Authors:** Jing Cai, Bodou Zhang, Yuqi Li, Wanfang Zhu, Toshihiro Akihisa, Wei Li, Takashi Kikuchi, Wenyuan Liu, Feng Feng, Jie Zhang

**Affiliations:** 1 School of Traditional Chinese Pharmacy, China Pharmaceutical University, Nanjing 210009, China; 3220020316@stu.cpu.edu.cn (J.C.); 1821020421@stu.cpu.edu.cn (B.Z.); 3219020456@stu.cpu.edu.cn (Y.L.); akihisa_toshihiro@yahoo.co.jp (T.A.); fengfeng@cpu.edu.cn (F.F.); 2 School of Pharmacy, China Pharmaceutical University, Nanjing 210009, China; 3119010060@stu.cpu.edu.cn (W.Z.); liuwenyuan@cpu.edu.cn (W.L.); 3 Research Institute for Science and Technology, Tokyo University of Science, Chiba 278-8510, Japan; 4 Faculty of Pharmaceutical Sciences, Toho University, Chiba 274-8510, Japan; liwei@phar.toho-u.ac.jp (W.L.); t.kikuchi@gly.oups.ac.jp (T.K.); 5 Jiangsu Food and Pharmaceutical Science College, Huaian 223003, China

**Keywords:** Epstein-Barr virus, vaccine, T cells, antibodies, oncogenic

## Abstract

The Epstein-Barr virus (EBV) infects more than 95% of adults worldwide and is associated with various malignant tumors and immune diseases, imparting a huge disease burden on the human population. Available EBV vaccines are imminent. Prophylactic vaccines can effectively prevent the spread of infection, whereas therapeutic vaccines mainly stimulate cell-mediated immunity and kill infected cells, thus curbing the development of malignant tumors. Nevertheless, there are still no approved EBV vaccines after decades of effort. The complexity of the EBV life cycle, the lack of appropriate animal models, and the limited reports on adjuvant selection and immune responses are gravely impeding progress in EBV vaccines. The soluble gp350 vaccine could reduce the incidence of infectious mononucleosis (IM), which seemed to offer hope, but could not prevent EBV infection. Continuous research and vaccine trials provide deep insights into the structural biology of viruses, the designs for immunogenicity, and the evolving vaccine platforms. Moreover, the new vaccine candidates are expected to achieve further success via combined immunization to elicit both a dual protection of B cells and epithelial cells, and sustainable immunization against infected cells at several phases of infection.

## 1. Introduction

**Figure 1:**
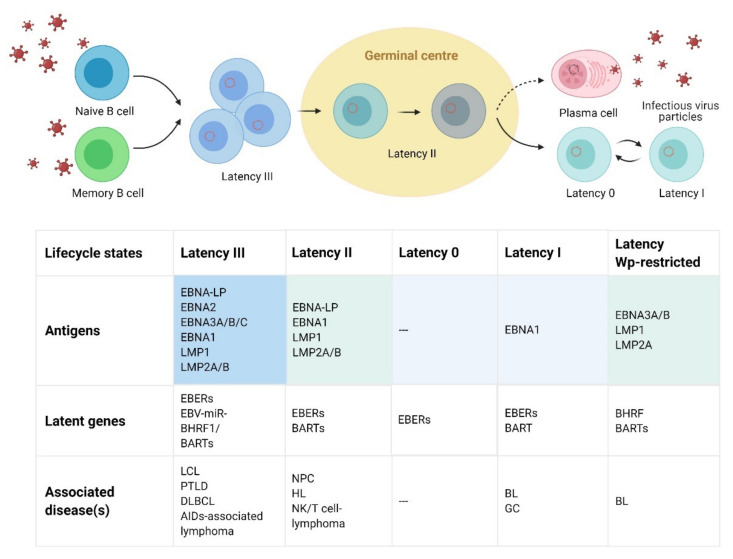
The phases of EBV infection in host cells and antigen expression. EBV in latent infection only expresses a minimal number of latent genes to maintain the stable presence of the virus’ genome in cells and to promote the survival of infected cells while minimizing host immune recognition. According to the protein expression patterns, three latency phases have been identified, and the types and numbers of latent genes expressed by each phase differs. Latency III is the most elaborate viral expression phase (EBERs, EBNA-LP, EBNA1, EBNA2, EBNA3A–3C, LMP1, LPM2A, and LMP2B). It is commonly observed in cells in a highly clonal proliferative state, such as newly infected B cells or EBV-immortalized lymphoblastoid cell lines (LCLs). This phase is associated with the post-transplant lymphoproliferative disorder (PTLD), the spread of large B cell lymphoma (PT-DLBCL) after EBV transplantation, and AIDS-associated diffuse large B cell lymphoma. Latency II is more limited in protein expression (EBERs, EBNA-LP, EBNA1, LMP1, LMP2A, and LMP2B) and is associated with nasopharyngeal carcinoma (NPC), PT-DLBCL, Hodgkin’s lymphoma (HL), and natural killer (NK)/T cell lymphoma. Latency I, the strictest latency phase (EBERs and EBNA1), can be found in proliferating memory B cells and is associated with post-transplant Burkitt lymphoma (BL) and gastric carcinoma (GC). Latency 0 is commonly in resting memory B cells without the expression of viral protein components. Moreover, the Wp-restricted latency phase (EBNA3A/3B, LMP1, and LMP2A) is associated with BL. In addition, non-coding RNA transcription of EBV in the state of latent infection exists. Figure created with BioRender.com.

The Epstein-Barr virus (EBV), also known as human herpesvirus 4 (HHV4), is a large double-stranded DNA (dsDNA) virus that belongs to the gammaherpesvirus subfamily [1]. EBV is the first identified oncovirus [2,3], with the most potent host-cell-transforming ability among all infectious disease pathogens in vitro, infecting approximately 95% of adults worldwide [4]. Most EBV infections occur in infants, establish a lifelong latent infection state, or reactivate into lytic replication under certain circumstances [5,6] (Figure 1). EBV induces the hyperactivation of T cells in adolescents and young adults, resulting in immune dysregulation. Primary EBV infection [7,8] may cause infectious mononucleosis (IM) [7,8], and the virus-induced overactivation of bone marrow cell cytokines may lead to hemophagocytic lymphohistiocytosis (HLH) [9]. In the later stages of infection, due to excessive immune responses, multiple autoimmune diseases arise, including multiple sclerosis (MS) [10], Sjogren syndrome (SS), systemic lupus erythematosus (SLE), dermatomyositis, rheumatoid arthritis (RA), inflammatory bowel disease (IBD), and type 1 diabetes mellitus (T1DM) [11]. EBV is also associated with various diseases and tumors, such as Burkitt’s lymphoma [12,13], post-transplant lymphoid-promoting disease (PTLD), diffuse large B cell lymphoma (DLBCL), Hodgkin’s lymphoma (BL) [14], nasopharyngeal carcinoma (NPC) [15], and 10% of gastric carcinomas (GC) [16,17]. The World Health Organization (WHO) has listed EBV as a class I oncogenic virus [18,19], with approximately 200,000 new cases of tumors directly associated with EBV annually worldwide [20].

The most effective way to control EBV prevalence is an EBV vaccine that should be safe, effective, and cost-effective. Considering the complex life cycle of EBV, some attempts have been made using EBV envelope proteins, latent antigens, and early lytic antigens. Unfortunately, there are still no licensed EBV vaccines after more than 50 years of effort [21,22]. This review aims to retrospect past and current vaccine progress, highlight knowledge gaps, and propose new directions and novel strategies for developing efficacious EBV vaccines.

## 2. EBV Virologic Features and Lifecycle

EBV is a dsDNA virus [23,24,25] with a diameter of approximately 170 nm, and consists of three basic parts: the lipid bilayer envelope, the pseudo-icosahedral nucleocapsid, and the middle pleomorphic tegument compartment [26,27,28]. The EBV 172-kb dsDNA genome is enclosed in the pseudo-icosahedral nucleocapsid. The pleomorphic tegument compartment in the middle contains 20–40 different viral proteins, and the viral glycoprotein at the lipid bilayer envelope is responsible for host recognition and membrane fusion [25,27,29].

EBV is mainly transmitted through saliva. After transfer across the mucosal epithelium, the virus infects B cells in secondary lymphoid tissues, such as the tonsils [8,30]. After infection, the virus injects the viral genome into the host cell nucleus through nuclear pore complexes, and the linear genome is circularized into the episomal form through terminal repeats [31]. During the primary infection, immature B cells are infected with EBV and then express EBV nuclear antigens, such as EBNA1, EBNA2, EBNA3A–EBNA3C, EBNA-LP, latent membrane protein 1 (LMP1), and LMP2, as well as EBV-encoded small RNAs (EBERs) and microRNA that activate latent B cells and encourage them to proliferate and transform (latency III) [32]. Alternatively, the virus stimulates the infected memory B cells to differentiate directly into latency 0. The virus can downregulate its immunogenic proteins and thus survive in B cells. B cells infected with EBV enter the follicle, amplify, form germinal centers, and express only three viral proteins (EBNA1, LMP1, and LMP2) (latency II). Then, they leave the lymph nodes and only express EBNA1 to separate the viral genome from the cell genome (latency I). When EBV-infected cells enter the peripheral blood, they shut down all of the viral genes (latency 0 or latency program). Hence, such EBV-infected resting memory cells are immune to the attack by the host’s immune system and could serve as sites for long-term viral persistence [16,33]. After reactivation into the lytic phase, the EBV genome is amplified up to 1000-fold by the viral replication machinery [34]. The immediate–early lytic proteins Zta and Rta are expressed [35], thereby activating transcription factors, regulating the expression of the early lytic proteins BMRF1, BALF1, and BHRF1, and promoting the replication starting point Ori-lyt to trigger EBV genomic DNA replication. Structural proteins are then expressed, such as the matrix protein, capsid components, and envelope proteins. The viral genomic DNA produced by replication and the newly synthesized viral structural proteins are assembled into new infectious virus particles that are finally released outside the cell through exocytosis or cell lysis [34,36].

## 3. Immune Responses to EBV Infection

### 3.1. Humoral Immune Responses

Primary EBV infection triggers a humoral immune response in the majority of infected individuals. B cells participate in the adaptive immune response and produce specific antibodies. The induction of immunoglobulin (Ig) M antibodies against viral capsid antigen (VCA) occurs in the early stage of infection and is maintained for weeks to months. After this, they switch to anti-VCA IgG antibodies that usually peak at two to four months after infection. The antibodies then decline and could last for a lifetime [37,38]. After the primary infection subsides, non-neutralizing targets, such as immediate–early and early antigens (BZLF1, BMRF1, BHRF1, BLRF2, BFRF3, gp42 [39], and EBNA 1, 2, and 3 [40]), appear. B cells also produce anti-EBNA1 IgG antibody and IgG/IgA antibodies against glycoprotein 350 (gp350) [39,41,42]; the gp350 antibody prevents EBV from binding to the CR2 receptor of B cells, limiting EBV transmission and reinfection [43,44,45]. In the latent infection phase, antigen-specific T cells, such as EBNA3A, -B, and -C, expand in vivo. The T cell function is inhibited in some patients with bone marrow suppression or organ transplantation who are prone to EBV-related lymphoproliferative diseases after transplantation [46,47].

### 3.2. Cellular Immune Responses

Several subsets of innate human lymphocytes target different stages of EBV infection. CD8+ T cells play an essential role in the adaptive cellular immune response. IM symptoms are thought to be caused by the increase in CD8+ T cell-related activities. Studies in both immunocompromised transplant patients and individuals with a primary immunodeficiency have demonstrated the critical role that CD8+ T cells play in controlling the outgrowth of EBV-transformed B cells [48]. They mainly recognize lytic EBV antigens expressed during the immediate–early stages (e.g., BZLF1 and BRLF1) and early stages of lytic infection (e.g., BMLF1 and BMRF1, BALF2, and BALF) [49] to effectively control EBV infection and lymphoproliferative diseases after transplantation, especially in the early stage of lytic infection. After IM subsides, the response to the lytic phase antigen shrinks, usually accounting for 0.2–2% of the total number of CD8+ T cells in latent infected individuals. In contrast, the response of T cells to the latent phase antigen peaked after the remission of acute IM symptoms, typically accounting for 0.05–1% of the CD8+ T cells [50,51,52]. Antigen-specific CD8+ T cells are mainly stimulated by EBNA1 and other latent proteins. EBNA1 inhibits recognition by CD8+ T cells by inhibiting the pre-mRNA processing of the primary EBNA-1 transcript and inducing the retardation of self-translation. EBNA1 has also been shown to be protected from proteasomal degradation, thus achieving immune evasion [53,54,55].

CD4+ T cells also play an essential role in controlling EBV infection. EBV-infected B cells widely express MHC-II molecules and activate CD4+ T cells. EBV-specific CD4+ T cell responses are readily identifiable against soluble lytic antigens (BZLF1, BMLF1, and BCRF1), the envelope components (gp350 and gp110) [56,57,58,59,60], and the potential latent antigens (EBNA 1, 2, 3C, and LMP2) [48]. CD4+ CTLs recognize antigens in EBV lytic/latent phases and kill infected B cells and LCLs through Fas/FasL interactions [61]. It often takes several months after infection for EBNA1 to stimulate an immune response by CD4+ T cells, explaining the delayed emergence of anti-EBNA1-IgG. Moreover, many CD4+ T cells that are specific for soluble antigens are perforin-positive and cytotoxic, suggesting that these cells may play a direct role in controlling viral replicative damage [48].

Similar to CD8+ T cells, NK cells expand significantly during IM [62,63]. NK cells preferentially recognize lytic EBV-replicating cells [64,65,66] and identify EBV-infected B cells that are not matched to MHC class I to control EBV infection [67]. Therefore, NK cells are preferred for targeting lytic EBV replication. Targeting other stages of EBV infection in the allogeneic environment could also be beneficial. Vγ9Vδ2 T cells are elevated in EBV-positive children (25–50%) [67]. They preferentially recognize EBV latency I-infected B cells, and, once activated, Vγ9Vδ2 T cells can also target other EBV latency phases, including latency III carrying EBV-transformed LCLs. Vγ9Vδ2 T cells complement NK cells by recognizing potential EBV infection [7]. A combination of two cytotoxic innate lymphocyte subsets may be beneficial for targeting EBV infection. NK T cells have a limited response to latency phase cells but preferentially recognize latency II. It is associated with extranodal NK/T-cell lymphoma-nasal type and aggressive NK-cell leukemia [68]. The immune control of EBV infection depends on these cytotoxic lymphocyte subsets that can be stimulated by an EBV-specific vaccine targeting different EBV-positive malignancies.

## 4. EBV Transmission: Extensive and Rebellious

The content of EBV DNA in the saliva of primary infected persons may be very high and persist for months, supporting the hypothesis that EBV infection in adolescents is primarily caused by deep kissing [69,70,71]. Besides sexual intercourse [72], the exchange of blood products, such as hematopoietic cell transplantation [73] and solid organ transplantation [74], can also promote EBV transmission and even cause IM [75,76,77]. However, the exact incidence is uncertain, and the risk does not appear to be high. A large number of EBV infections occur in infants and children, and the mechanism is still unclear. A reasonable hypothesis is that these infections originate from close contact with parents, relatives, or caregivers. EBV is regularly released into the oral secreta of the “carriers” [78,79]. In South Africa, children use saliva to wash their faces, gargle, and eat pre-chewed food [80], all of which could increase the likelihood of infection with EBV.

The prevalence of EBV infection in adults worldwide is as high as approximately 95%, making it difficult or even impossible to limit exposure to EBV to contain the spread of primary EBV infection. Protection against EBV infection is imminent. An EBV vaccine would be the most effective way to control the EBV epidemic scientifically, but there are still no approved EBV vaccines after decades of effort. Nevertheless, it is still urgent to further promote the development of EBV vaccines.

## 5. Challenges in EBV Vaccine Development

### 5.1. Immunization Surveillance and Efficacy Assessment

Preventive and/or therapeutic EBV vaccines are designed to induce humoral and/or cellular immune responses to enhance treatment-related immune reactions. The complete prevention of EBV infection seems an elusive goal. If a vaccine provides only temporary immune protection or delays primary EBV infection, the virus will eventually go into uncontrolled lytic replication and could contribute to the occurrence of IM [8,81]. Thus, establishing or maintaining immune control against asymptomatic persistent EBV infection is the goal of EBV prophylactic vaccination [82,83]. The major problems with the development of a therapeutic EBV vaccine are the low incidence and sample sizes (fewer than 50 per 100,000 individuals [15]) in malignancies, as well as the inaccurate long-term assessment of vaccine efficacy due to the established immunosuppressive mechanism in vivo.

Since Epstein first proposed a vaccine in 1976 [21,22], several preventive and therapeutic vaccine strategies have been evaluated in clinical trials [84,85]. However, none have achieved the desired immune efficacy. The key index of preventive immunity is the neutralizing antibody (nAb) titer [86,87]. Neutralizing antibodies can effectively block the attachment of the virus to host receptors and the fusion between them, and a higher titer theoretically indicates that the antiviral infection effect is very evident. However, the value of this index in predicting the incidence of malignancy remains unclear [88,89]. The T cell response is also a critical component limiting EBV latent infection and the adaptive immune response [90,91,92,93,94,95]. Other EBV antigens need to be used in vaccine combinations to improve the vaccine efficacy, especially those with specific CD4+ and CD8+ T cell responses. In vitro studies have shown that CD4+ T cells specific for gp350 and other virus particles could recognize B cells very soon after EBV infection. Other EBV antigens, such as EBNA2, EBNA-LP, and LMPs, can be used as targets for CD8+ T cells. EBV vaccines against multiple immune responses to viruses can provide more comprehensive protection and control the immunological surveillance of primary infection and EBV-associated malignancies. Some knowledge gaps still need to be addressed, and further research is required to determine the role of the vital immunological components of EBV immunity establishment, including the role of B cell immunity during primary infection [96,97,98], T-cell-mediated immunity during latent infection [97], and the implementation of more relevant target antigens against EBV immunity [99].

### 5.2. Adjuvant Selection

Adjuvants modulate the immune efficacy of EBV vaccine formulations by enhancing the initial protection against primary infection and secondary protection against the reactivation or expansion of latent infection. Adjuvants, such as Freund’s adjuvant, lipid A, immune-stimulating complexes (ISCOMS), and aluminum hydroxide, have been used to formulate gp350 vaccines [100,101,102,103,104]. Some of them have shown a better immunization efficacy than those without adjuvants. More complex adjuvants have entered preclinical and clinical trials. The level of antibodies induced by gp350/aluminum hydroxide and 3-O-desacyl-4’-monophosphoryl lipid A (AS04) is higher than that of traditional gp350/ALUM adjuvants [105], suggesting that an improved vaccine adjuvant combination might enhance the efficacy of an EBV vaccine. Therefore, vaccine combinations need more potent adjuvants to pre-stimulate immune recognition and improve both humoral and cellular immune responses.

### 5.3. Appropriate Animal Models

A suitable animal model can provide a potential and critical platform for evaluating immune mechanisms and can target antigens for the prevention/control of EBV infection after vaccination. Meanwhile, EBV infection is highly species-specific, resulting in a lack of sufficient animal models for preclinical evaluation and an accurate understanding of the clinical relevance of protection and uncertainties in ancillary choices. The early gp350 vaccine candidates were tested for safety and immunogenicity using non-human primate (NHP) cotton-top tamarins and common marmosets (*Callithrix jacchus*) that have a high degree of homology to human genes [106,107]. However, these NHPs are critically endangered and are no longer available. The rhesus macaque is an applicable animal model for evaluating EBV-specific T cell responses [108,109,110], while the virus cannot effectively infect its B cells [111]. Moreover, the rhesus macaque is sensitive to LCV (i.e., rhLCV), which has a high genome sequence similarity to EBV [112]. When challenged with rhLCV, protected rhesus monkeys showed higher plasma anti-EBV gH/gL neutralizing antibody AMMO1 neutralization activity. This result showed that the soluble LCV gp350 vaccine in rhesus monkeys reduced both EBV infection and long-term viral load incidence [113].

Rabbits can also be used as potential animal models to test the efficacy of EBV vaccines against primary and persistent infection [114,115,116,117]. After infection through the veins or mucous membranes, the anti-EBV VCA titer and EBV DNA level can be detected in most rabbits’ blood. However, the infection status is unstable, with only some rabbit spleen samples showing positivity for EBERs, LMP1, or EBNA, and few rabbits maintaining EBV-positive infections.

The mouse immune model is mainly used to evaluate the titer of serum and neutralizing antibodies [118,119,120]. A severely immunocompromised mouse model with a human immune system was established by transferring CD34+ hemopoietic stem cells [121,122,123,124]. After vaccination in a humanized mouse model, reconstructed human immune components (such as T cells, B cells, and dendritic cells [DCs]) are crucial mediators for inducing adaptive immune responses and promoting effective immunity. A humanized mouse model can also evaluate the protective effect of AMMO1, with the results showing that antibodies can effectively inhibit EBV infection [125].

### 5.4. The Complexity of EBV Infection and Vaccine Design

EBV can infect various cell types, but only details regarding B cell and epithelial cell infection are known. EBV infects B cells and epithelial cells via a different set of glycoproteins (i.e., gp350, gp42, gH, gL, and gB). These glycoproteins are excellent vaccine targets [126,127,128,129]. However, it is unclear whether EBV vaccines should target multiple glycoproteins to effectively block EBV infection in various cell types [128,129]. Moreover, EBV can infect T cells [130,131] and NK cells [132] through as-yet undefined mechanisms, suggesting that EBV vaccines cannot currently be properly designed to prevent the infection of all susceptible cell types. Structural proteins can be recognized not only by neutralizing antibodies but also by T cell reactions. Infected B cells are recognized by envelope (e.g., gp350, gH, and gB)- and tegument (e.g., BNRF1)-specific CD4+ T cells [133,134,135]. Thus, vaccines containing structural antigens, in addition to producing neutralizing antibodies, may also trigger protective T cell responses that target the virus and newly infected cells in early infection.

Infected B cells are recognized to varying degrees by latent (e.g., EBNA2 and EBNA-LP) and lytic (e.g., BHRF1) protein-specific CD4+ and CD8+ T cells [133]. For example, EBNA2 MHC-I-restricted epitopes are most effectively-identified early, indicating that the latent protein EBNA2 is a promising vaccine target. Potential antigens are present in EBV-associated diseases and malignancies, making it possible to reduce the disease burden of EBV with a latent protein vaccine [136,137,138]. Vaccines that induce latent protein-specific responses may help to target EBV-infected cells before and after transformation. More than 80 lytic genes are expressed during the EBV lytic phase [139]. Lytic-replicating cells express many antigens, and T cells often recognize immediate–early, early, and late antigens [51,140,141] and subject them to immune control. In addition, hydrolytic-replicating cells display viral glycoproteins on their surfaces, making them a potential target for antibody-dependent cytotoxicity (ADCC) [142,143,144,145]. Thus, lytic-replicating cells are subjected to cellular and adaptive immune responses.

In summary, EBV-associated diseases and malignancies may be reduced by vaccination against EBV, but this may not achieve sterile immunity [20]. Live-attenuated vaccines are not suitable for EBV since live-attenuated herpesvirus may persist in infected individuals [146]. Further EBV vaccine exploration needs to take into account the complexity of EBV infection, such as the mechanisms of infection in different cell types, direct cell-to-cell [147,148], and the multiple antigens that predominate during EBV’s life cycle, in order to ensure that the EBV vaccine is effective and safe.

## 6. Progress: Where Are We Now?

### 6.1. Envelope Protein Vaccines: Neutralizing Antibodies Elicited

**Figure 2:**
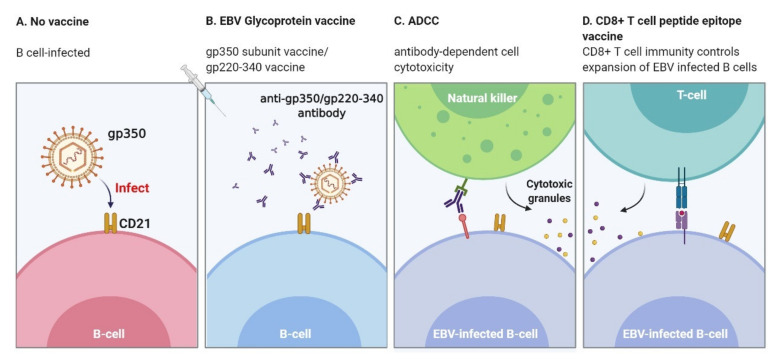
Mechanism of prophylactic vaccine action: (**A**) EBV virions bind to CD21 on B cells through gp350 and interact with HLA class II molecules to trigger gB-mediated viral–host membrane fusion within endosomes; (**B**) EBV membrane antigens are used as immunogens, and trigger neutralizing antibodies targeting virus particles to prevent the infection of host B cells; (**C**) the binding of antibodies with glycoprotein on the surface of infected cells can be recognized and eliminated by NK cells; (**D**) EBV-infected cells are recognized by T cells, which release cytotoxic granules and trigger the apoptosis of infected cells. Figure created with BioRender.com.

By infecting the epithelial cells in the oropharynx, EBV is transported to B cells through blood vessels in the epithelial tissue and enters the latent infection phase [149]. At least five viral envelope glycoproteins (gp350, gB, gH, gL, and gp42) and three cellular proteins (CD21, HLA, and integrin) are required for EBV to enter B cells effectively. Gp350, the main component of the viral membrane, binds to the complement receptor type 2 (CD21) and type 1 (CD35) of B cells. The gp42 then forms a stable complex with gH/gL and binds to human leukocyte antigen (HLA) class II to facilitate the endocytosis of the virion, and gB triggers the membrane to fuse with endocrine vesicles [43,150,151,152]. Other observations also show that gp350 is a target for ADCC [144,153], as well as for CD4+ T cell [59,60] and CD8+ T cell responses [154]. Clinical trials have proven the safety and biological activity of the gp350 vaccine [105,155]. The EBV membrane antigen BNLF-1 MA (gp350/220) is also key to establishing EBV latent infection. Gp350/220 initiates the attachment of EBV to a susceptible host that expresses CD21 and/or CD35 [156]. The binding is further strengthened by the gp42 envelope protein interacting with MHC class II [134,157]. It is one of the antigenic candidates of EBV-preventive vaccine exploration (Figure 2). The main neutralizing antibody is the monoclonal antibody 72A1. It can block the EBV infection of B cells in vitro [158]. Other studies have shown that the EBV glycoproteins gB, gH, gL [129,159,160], and gp42 [161] can also induce the production of protective neutralizing antibodies. Two prophylactic vaccines that induce neutralizing antibodies are currently under study [161,162].

EBV membrane antigens are often used as immunogens [163]. Gp350, due to its abundance, is the core antigen of the EBV vaccines currently under development [164,165,166,167,168]. The first clinical trial of the vaccinia construct expressing the EBV membrane antigen gp220–340 in China in 1995 demonstrated that gp220-340-specific antibodies could be elicited in both seronegative and seropositive children [169] (Table 1). The EBV live vaccine vector has shown some effectiveness. However, the sample size was too small to conclude its immune efficacy. Due to the safety concerns of live vaccinia vectors, no further research has been carried out on this method [170].

The recombinant subunit proteins expressed in Chinese hamster ovary (CHO) cells have been used for making a gp350 candidate vaccine induce gp350 neutralizing antibodies successfully in rabbits [167]. This vaccine has been used in four clinical trials in humans and is still under development. In a phase I trial of the gp350 subunit vaccine [105], the safety and immunogenicity of three doses of the intramuscular vaccine containing 50 mg of gp350 was evaluated in both EBV seropositive and seronegative adults. After immunization, all of the 22 seronegative subjects had specific immune responses to the vaccine and produced anti-gp350 antibodies, and the antibodies were more likely to be neutralized in subjects who received the vaccine in an adjuvant system 04 (AS04). In another phase 1/2 study, a total of 138 adult subjects aged 18–25 who were serologically negative or positive for EBV were randomized to receive the gp350 vaccine in an aluminum salt adjuvant, AS04, and no adjuvant [105]. All of the subjects developed an antibody titer for gp350 ELISA, and the vaccine inoculated with the AS04 adjuvant produced a higher neutralization titer. However, a suspected vaccine-related adverse event occurred. A subject positive for EBV antibodies who received the second dose of the AS04 adjuvant gp350 subunit vaccine was hospitalized with a significant autoimmune reaction involving the central nervous system and multiple joints.

A placebo-controlled, double-blind, phase II clinical trial was conducted to test the safety, immune response, and efficacy of the AS04 recombinant gp350 vaccine. A total of 181 adolescent/adult subjects aged 16–25 years with a seronegative mononuclear disease risk were investigated [155]. The results showed that this vaccine had a significant effect on clinical disease, whereby 98.7% (76/77) of the vaccine recipients showed gp350 seroconversion lasting six months. This vaccine was 77.9% effective in preventing IM caused by EBV infection, and elicited anti-gp350 antibodies that were maintained in vivo for over 18 months. Despite these encouraging results, no evidence of protection against asymptomatic infection was found; 13 out of 90 vaccine recipients (14.4%) were infected, compared to 18 out of 91 placebo subjects (19.8%).

Finally, a phase I clinical trial of the recombinant gp350 subunit vaccine with an alumina hydroxide adjuvant was conducted in 16 EBV-negative children with chronic renal insufficiency who were awaiting renal organ transplantation [171]. Thirteen of the sixteen subjects produced gp350 antibody responses after inoculation, but only four subjects showed EBV-neutralizing antibody responses. These results show that the vaccine has good biological activity and immunogenicity, but a control group is unavailable for evaluating the curative effect. Additional doses of the vaccine and/or more effective adjuvants are still needed to shorten the time from the initial vaccination to organ transplantation in order to reduce the viral load of EBV in patients after transplantation, and to lower the risk of developing PTLD after transplantation immunosuppression.

The preventive vaccine with the viral envelope glycoprotein gp350 as the target antigen can effectively reduce the occurrence of IM, but it is not adequate for asymptomatic or specific EBV-infected persons [105,155]. This suggests that vaccination against the gp350 protein alone could not lead to complete protection. Therefore, improving antibody titers through adjuvant modification, the delivery of polymeric forms of gp350, or an increase in the diversity of neutralizing antibodies in combination with multiple proteins, such as gB, gH, gL, and gp42, in order to prevent EBV infection, may be a future direction [105,125].

### 6.2. Protein Polymers and Nano-Vaccines Enhance the Response of Antibodies

Cui et al. designed an EBV gH/gL trimer protein, a gH/gL monomer protein, a gB trimer protein, and a tetrameric gp350-based vaccine [119,160,172] to induce neutralization antibodies after immunizing rabbits [160]. The tetramer used Gly4Ser1 3 as the linker for two identical gp350 sequences, and the self-binding action of the leucine zipper sequence of Saccharomycete GCN4 enabled the gp350 dimer containing the CD21 binding site to form the tetramer protein [171]. This tetramer gp350 vaccine enhances gp350-specific T cell immunity and improves virus neutralization titers. Additionally, a high IgG titrant and strong anti-gp350 CD4+ T cell response have been detected in mice [114] inoculated with the GLA/SE adjuvant gp350 vaccine.

Kanekiyo et al. constructed nanoparticles based on EBV gp350^1–425^ [44] that are not easily cleared by the immune system due to the lack of mucin regions in the 1–425-amino-acid-residue gp350 glycoprotein. The authors tested this vaccine construct in mice and primates. The results have shown that the level of nanoparticles, after antigen immune protein-neutralizing antibodies, is higher than that for gp350^1–859^ by approximately 10–100 times [44]. The application of nanotechnology provides a new method for developing an EBV-preventive vaccine. Notably, vaccinated animals did not become infected with EBV. However, it is unclear whether these neutralizing antibody titers would inhibit EBV infection in vivo.

In addition, a more efficient peptide vaccine containing a neutralizing epitope of the EBV envelope glycoprotein was established by epitope mapping. The investigators focused on inducing immune responses against the dominant neutralizing epitopes of gp350 and other envelope proteins through introducing strong ionic, electrostatic, or hydrogen bonding to the neutralizing region of mAb 72A1 [173] with computer modeling [174]. These biomimetic peptides blocked the interaction of the 72A1 antibody and gp350 in mice and elicited an antibody response.

To elicit a more comprehensive humoral immune response, the neutralizing anti-gH/gL antibody AMMO1 [129] that targets the EBV fusion machinery proteins, was isolated from memory B cells to produce a vaccine consisting of the envelope glycoprotein and viral fusion proteins. It showed a potent inhibition of B cell and epithelial cell infections in vitro [128]. A nano-vaccine using tannic acid (TA) and EBV-related tumor protein antigens, with interferon-α (IFN-α) or CpG as adjuvants [175,176], can significantly induce immune activation by targeting lymph nodes. Moreover, combining it with anti-PD-L1 resulted in a marked decrease in tumor size and prolonged the survival time of tumor-bearing mice [175].

### 6.3. CD8+ T cell Peptide Epitope Vaccine

The prophylactic EBV vaccine can also control the EBV transmission by inducing cell-mediated immune responses, such as specific CD8+ T cell responses to EBV latency EBNA proteins [177]. Elliott et al. made a CD8+ T cell peptide epitope-based vaccine that used FLRGRAYGL, an HLA-B*0801 restriction antigen epitope encoded by EBNA 3A, which can induce a specific CD8+ T cell immune response to EBNAs [125,178]. The vaccine was tested in a randomized, single-blind, placebo-controlled, single-center phase I clinical trial in 14 EBV-seronegative young adults [178]. The subjects received either EBNA-3A peptides with tetanus toxoid as an adjuvant with an oil-in-water emulsion (Montanide ISA 720) or the placebo at two-month intervals. Most of the subjects (8/9) with peptide-specific CD8+ T cell responses were shown to be effectively protected. Although this study was too small to determine the effectiveness of T cell immune induction in preventing primary symptomatic infection, it does demonstrate the feasibility of inducing cell-mediated immunity in health vaccinators by targeting EBV latency antigens.

With a deeper understanding of T cell immunity in the control of EBV infection [60,179,180,181,182], the application of latent- or lytic-phase proteins as vaccine antigens has shown great potential in preclinical studies [183,184,185,186,187]. Several vaccine applications of latent- or lytic-phase proteins as vaccine antigen therapies expressed in malignancies are being developed in preclinical and early clinical trials [120,122,188,189].

### 6.4. DC Vaccines

The first clinical trial to evaluate the targeting of LMP antigens or EBNA1 was a vaccine trial based on dendritic cells (DCs). DCs are the most potent professional antigen-presenting cells that can activate resting T cells. DCs loaded with target peptides or proteins after culture and amplification in vitro can induce specific immune responses. In a phase I trial of therapeutic vaccination in 16 NPC patients [90], after four peptide-pulsed monocyte-derived DCs injections, nine patients had enhanced antigen-determine-specific CD8+ T cell responses that were associated with a slight decrease in serum EBV DNA levels. Tumor regression was observed in two of the nine patients [125]. A similar study of LMP2-pulsed DCs by Lin et al. showed an increase in the LMP2 response [190]. Although this was not associated with a reduction in peripheral EBV load, the delayed hypersensitivity (DTH) did lead to a decrease in the EBV load. An alternative DC vaccine is made by transducing DCs with an adenoviral vector encoding the truncated LMP1 (Ad–Δ LMP1–LMP2) [91]. Nine out of twelve NPC patients developed DTH responses in this phase II study of metastatic NPC-positive individuals. No change in the frequency of peripheral LMP-specific T cells was detected; three patients did show clinical responses, including partial remission (lasting 7.5 months), and two showed disease stabilization (lasting 6.5 and 7.5 months). These observations suggested that LMP-specific T cell responses may be induced after DC vaccination. However, the widespread use of DC vaccines is unfeasible, as the costs associated with personalized DC preparation can be high. A more appropriate approach might be to deliver the immunological determinants of LMP1, LMP2, and EBNA1 via viral vectors, or to formulate them in conjunction with adjuvants already licensed for use in humans.

### 6.5. Viral Vector Vaccines

Recombinant viral vector vaccines are live viruses that offer advantages over traditional vaccines. First, they can induce a wide range of immune responses [191,192] to effectively clear virus-infected cells and tumor cells, especially in CD8+ cytotoxic T cell (CTL) responses. Second, the body’s natural immunity against live viruses has the effect of enhancing protective immune responses through the expression of a series of pathogen-associated molecular patterns (PAMPs) that elicit inflammatory responses. Third, viral vector vaccines have high gene transduction efficiencies [193]. Poxviruses, adenoviruses, and yellow fever viruses were developed as vaccine candidates [191]. However, the pre-existing immunity from the human viral vectors needs to be considered. Vaccinia virus and adenovirus are among the most widely used viral vectors, mainly because of their ability to induce antigen-specific T cell responses.

The first EBV vaccine tested in humans used a live vaccinia recombinant vaccine expressing the EBV membrane antigen BNLF-1 MA (gp350) [169]. Although there was no significant change in the EBV infection between vaccinated and unvaccinated adults, EBV infection rates among vaccinated infants were reduced. This vaccine platform is no longer accepted due to the risk of adverse reactions [170].

A safer alternative viral vector is the multiplication-incompetent attenuated pox viral vector of the modified vaccinia virus Ankara (MVA) [194,195]. It has been used in vaccine clinical trials against other pathogens and can significantly induce T and B cell responses in human subjects with good safety. The recombinant EBV vaccinia Ankara virus strain vector vaccine (MVA-EL) was designed to encode a fusion protein of the 3’ half of the EBNA1 gene and the full-length LMP2 gene [92,93]. Taylor et al. used the MVA-EL vaccine, which expressed partial EBNA1 and LMP2 proteins simultaneously in a phase I clinical trial [93]. In this study, EBV-positive NPC patients showed that the vaccine could effectively induce the expansion of EBNA1- and LMP2-specific CD4+ and CD8+ cells in the peripheral blood lymphocytes of EBV-seropositive patients. Eight of the fourteen patients had elevated levels of CD4+- and CD8+-specific T cell immune responses. Immuno-phenotypic analysis showed that EBNA1- and LMP2-specific CD4+ and CD8+ T cell differentiation and functional diversification could be induced by vaccination, proving that the vaccine has good immunogenicity and safety. The updated phase I clinical trial is designed to determine the safety, tolerability and immunogenicity of the extended schedule vaccination with MVA-EBNA1/LMP2 in EBV+ NPC patients [NCT01800071]. The same MVA-EL vaccine was tested in phase I clinical trials in EBV-positive NPC patients in Hong Kong [92], and was well-tolerated with no dose-limiting toxicity. Specific T cell responses were increased in 15 of the 18 patients, and the number of specific CTLs in the peripheral blood was increased by three to four times. The immune remission of patients lasted more than 12 weeks, but a small number of subjects experienced mostly grade 1 adverse reactions after receiving the vaccine. Only three patients experienced grades 2–3 adverse events.

Considering the complexity of cell vaccine methods, the adenovirus vector encoding the EBV antigen could be considered for use to elicit pre-existing EBV-specific immunity. Adenovirus vectors encoding LMP2 are also in clinical development. Si et al. conducted a phase I clinical trial in NPC patients using a recombinant adenovirus vaccine carrying EBV LMP2 (rAd5-EBV-LMP2) [95]. Subjects were EBV IgA/VCA serum-positive with stable advanced NPC treated under routine chemotherapy. The results showed that CD3+ and CD4+ cells in the peripheral blood of immunized patients were dose-dependent. With no severe adverse reaction, this proves that the recombinant adenovirus vector vaccine has adequate safety. After two years of follow-up, 83.3% of the patients were non-progressive after vaccination, as compared to 80% after conventional chemo-radiotherapy.

DCs or EBV-transformed B lymphocyte lines could be infected in vitro with or without EBNA1 using vectors encoding LMP to expand the EBV-specific T cell response [196]. Hartlage et al. inoculated SCID mice (injected with human peripheral blood mononuclear cells with an EBV-seropositive donor) with Zta-expressing adenovirus or DCs transduced by an adenovirus with empty vectors. Compared to the control group, mice that received DCs expressing Zta produced Zta-specific T cell responses and showed a delayed development of the lymphoproliferative disease of EBV [122].

### 6.6. Virus-Like Particles (VLPs)

Virus-like particles (VLPs) can induce neutralizing antibody targets. Compared to recombinant antigens, VLPs have a potential advantage because they can mimic the natural structure of the virus while not containing viral DNA [135]. Terminal repeats (TRS) were identified as packaging signals for EBV DNA [197,198], and the first generation of cell lines producing EBV VLPs was formed by removing TRS. EBV VLPs can bind human B and epithelial cells. Ruiss et al. prepared a viral material with a structure similar to EBV and obtained a cell line based on it [199]. The cell line has been genetically modified to contain viral proteins that are only essential for assembly and release. This material has a strong immunogenicity and can effectively stimulate the immune responses of CD4+ and CD8+ T cells in vivo. Pavlova et al. demonstrated that the deletion of BLFL1 and BRRF1A genes promoted DNA-free EBV VLPs with a certain immunogenicity [134]. Compared to the BFLF1/BFRF1A mutant EBV strain used as the wild type [134] that elicited comparable CD4+ T cell responses, the pathogenic potential of EBV VLPs was reduced. However, immune responses to EBV structural and disintegrated components were insufficient in generating an effective EBV vaccine. Moreover, Ogembo et al. found that human complement receptor 1 (CD35) can also be used as an EBV receptor, and constructed virus-like particles with EBVgp350/220 chimerism (VLP-subunit vaccine) [189]. The vaccine produced persistent neutralizing antibodies in mice. However, the serum antibody titer was slightly lower than the UV-EBV titer of the control group (EBV inactivated by 254 nm UV for 5 min), possibly due to the misfolding of the surface proteins of VLPs caused by the insertion of foreign genes that reduced the immunogenicity.

A chimeric VLP based on the hepatitis B core antigen (HBc149) has been designed [200]. All HBc149 proteins self-assemble into VLPs with gp350 epitopes displayed on the surfaces of spherical particles. The different orders of the three epitopes in the chimeric proteins induced different immune responses in Balb/C mice. Two constructs (149-3A and 149-3B) induced a high serum titer against the receptor-binding domain of gp350 and elicited neutralizing antibodies in immunized mice, which efficiently blocked EBV infection in cell cultures. Competition analysis has shown that the sera from these mice contained antibodies to a major neutralizing epitope recognized by the neutralizing mAb 72A1. HBc149 chimeric VLPs provide a valuable platform for presenting EBV gp350 antigens and offer a robust basis for developing peptide-based candidate vaccines against EBV.

### 6.7. Nucleic Acid Vaccines

To maximize the probability of T cells (including CD4+ and CD8+) recognizing viruses, overlapping PCR technology has been used to integrate the genes encoding for EBV EBNA1, LMP1, and LMP2. They have been inserted into adenovirus expression vectors to activate CD4+ and CD8+ T cells in NPC immunotherapy [196,201]. Li et al. reported that, after initial immunization with a DNA vaccine containing the LMP2 gene, the immunogenicity was enhanced compared with the LMP2 epitope peptide vaccine containing polypeptides. It binds to CTLs, T helper cells, B cells, and mouse-restricted CTL-binding poly-peptides, resulting in cellular immune responses [202].

Beyond this, the development of CRISPR/Cas9 genome-editing techniques are providing novel strategies for combating productive and latent EBV infections, such as viral genetic elements needed to target viral adaptability [203]. Lebbink et al. demonstrated that the virus could be almost eliminated from EBV-transformed cells with latent infection by simultaneously targeting the EBV genome with multiple guided RNA (gRNA) [204]. This provided a new way for developing therapeutic approaches for pathogenic human herpesvirus through new genomic-engineering techniques, such as antiviral drugs and other small molecules. Noh et al. targeted EBNA1 and used the transcriptional activation inducer E1TN to target the EBNA1 gene [205], inducing the gradual apoptosis of EBV-positive B cells, while leaving the EBV-negative cells unaffected. In addition, mRNA vaccines, as an effective, rapid, and universal vaccine platform, have attracted much attention in recent years, especially with the emergence of the COVID-19 mRNA vaccine. However, EBV mRNA vaccine development is still in its early stages. Moderna announced its ambitions for EBV mRNA vaccine development, with candidates for the major EBV glycoproteins gp350, gB, gH/gL, and gp42 [206].

## 7. Conclusions and Outlooks

For centuries, vaccines have been used to prevent and treat various diseases, and widespread vaccination has saved millions of lives by successfully eradicating smallpox and significantly reducing other infectious diseases [207,208]. Traditional vaccine formulations, such as subunit vaccines, attenuated live vaccines, and inactivated pathogens vaccines, that provide strong protection against many deadly diseases, are unsuitable for EBV [209,210]. The low levels of neutralizing antibody titers elicited by gp350 subunit vaccines are insufficient to induce adequate immune protection [154,170]. Future vaccines must adopt multipronged approaches capable of stimulating more than one arm of the immune system. Reasonable approaches involve improving adjuvants, protein–polymer formations, enhancing antibody diversity, or developing novel strategies, such as targeted B cell precursors that produce broadly neutralizing antibodies, novel viral vectors, and multi-vector sequential vaccines. The presence of viruses in EBV-positive tumor patients provides a target for tumor immunotherapy, and improving the level of specific CTLs is the focus of EBV therapeutic vaccine development. Viral vectors carrying the EBV gene, DCs, and other antigen-presenting cells loaded with EBV-related proteins or epitope polypeptides have been used to induce and activate the corresponding CTLs of patients and clear tumor cells. Meanwhile, passive immunotherapy includes directly activating patient-specific CTLs or modifies CTLs in vitro and infusing them back to the patient. Immunotherapy used in combination with clinical conventional radiotherapy and chemotherapy is expected to prevent the recurrence or metastasis of tumors.

The complexity of the EBV life cycle, the lack of suitable animal models, and our limited understanding of the immune response needed to prevent EBV infection have hindered the progress in vaccine development. There is still reason for cautious optimism that the numerous current clinical or preclinical developments for vaccine candidates will lead to further success, although this success may be modest and iterative. The valuable knowledge accumulated from numerous basic and translational science studies and vaccine trials has provided insight into the structural biology of viruses, immunogenic design, and novel vaccine delivery systems that may constitute effective vaccines. Further insights from humoral or cellular immunity levels may inspire immunotherapies targeting the pathology associated with different EBV infection processes. Multiple stimulations of the immune system require active efforts to seek combination vaccination methods in order to induce innate and adaptive immune responses. Gene editing and nanomaterial technology will also help to propel the development of an EBV vaccine.

## Figures and Tables

**Table 1:** Illustration of completed and documented EBV vaccine trials.

Vaccine	Year	Target Group	Immune Response	Result	Reference
Live recombinant virus gp350 vaccinia	1995	Nine children that were both EBV-seropositive and vaccinia-virus-seronegative	Vaccination boosted EBV-neutralizing antibody titers, but the target group was too small to prove efficacy	No vaccine efficacy	[169,170]
Recombinant gp350 vaccine produced in Chinese hamster ovary cells	2007	EBV-seropositive and seronegative adults	ELISA antibody titers to gp350 were detected; higher efficacy with MPL adjuvant	No vaccine efficacy	[105]
	2007	EBV-seronegative adults	ELISA antibody titers to gp350 were detected; neutralizing titers developed in 50–60% of persons; higher efficacy with alum adjuvant	One serious adverse event occurred; no vaccine efficacy	[105]
Recombinant gp350 vaccine	2007	181 EBV-negative teenagers/adult subjects	Neutralizing antibodies were detected; vaccine efficacy to prevent infectious mononucleosis by 78%, but no prevention of EBV infection	No serious adverse events were reported; prospects for prevention of Hodgkin’s lymphoma or MS. No further reports	[155]
Recombinant gp350 vaccine	2009	16 pediatric renal-transplant EBV-seronegative candidates	Poorly immunogenic, probably due to a low dose and weak adjuvant	The trial could not assess protection from PTLD	[171]
EBV peptide vaccine	2008	14 HLA B*801 EBV-seronegative young adults	A CD8+ T cell peptide vaccine was immunogenic with a hint of efficacy	No serious adverse events occurred; CD8+ T cell peptide vaccine: HLA restricted	[125,178]
EBV-specific HLA-A2-restricted DC vaccine	2013	16 human leukocyte antigen-A2 (HLA-A2)-positive NPC patients	Th1-specific immune responses were elicited, particularly in DTH test-positive individuals	The vaccine was well-tolerated; this vaccination is a promising treatment for EBV-related NPCs	[190]
Adenovirus ΔLMP1–LMP2 transduced DC vaccine	2012	16 metastatic NPC patients	Modified DC induced a low-level immune response	The potency of the current vaccine was too low for significant benefits in patients with extensive disease	[91]
AdE1-LMPpoly vaccine	2012	24 EBV-positive nasopharyngeal carcinoma (NPC)	Highly efficient in expanding antigen-specific T cells from patients with advanced recurrent or metastatic NPC disease, and these expanded T cells displayed high levels of functional capacity, as assessed by IFN-γ expression	The AdE1-LMPpoly vaccine was safe and well-tolerated and may offer clinical benefits to patients with NPC	[196]
Ankara vaccinia recombinant vector expressing EBNA-1 and LMP2	2013	18 EBV-positive NPC	CD4+ T cell responses to one or two vaccine antigens	MVA-EL was both safe and immunogenic. However, therapeutic efficacy has not yet been assessed. The highest dose is to be examined in phase II studies for clinical benefits	[92]
	2014	16 EBV-positive NPC	CD4+ T cell responses to one or two vaccine antigens	MVA-EL was safe and immunogenic across diverse ethnicities and thus suitable for use in trials against different EBV-positive cancers globally	[93]
Adenoviral vaccine of EBV-LMP2 (rAd5-EBV-LMP2)	2016	24 patients with advanced regional NPC	Failed to significantly affect peripheral CD8+ T cells	The rAd5-EBV-LMP2 vaccine was safe and well-tolerated, but has no vaccine efficacy	[95]

## Data Availability

The data presented in this study are available on request from the corresponding author.
